# Case Report: Oxytocin and Its Association With Psychotherapy Process and Outcome

**DOI:** 10.3389/fpsyt.2021.691055

**Published:** 2021-09-14

**Authors:** Ariella Grossman-Giron, Dana Tzur Bitan, Sigal Zilcha-Mano, Uri Nitzan, Shlomo Mendlovic, Hagai Maoz

**Affiliations:** ^1^Department of Behavioral Sciences, Ariel University, Ariel, Israel; ^2^Shalvata Mental Health Center, Hod Hasharon, Affiliated With the Sackler School of Medicine, Tel Aviv University, Tel Aviv, Israel; ^3^Department of Psychology, Haifa University, Haifa, Israel

**Keywords:** psychotherapy, outcome, oxytocin, affective disorders, case report

## Abstract

The potential of Oxytocin (OT) as a facilitator of psychotherapy has been previously acknowledged, nonetheless, randomized controlled trials thus far have not yielded conclusive results. One approach suggested to clarify empirical inconsistencies is to refine the study hypotheses and data collection process by utilizing an in-depth idiographic exploration of the investigated phenomena. In this case illustration we provide an in-depth analysis comparing two patients hospitalized in a closed psychiatric ward with depression and undergoing psychotherapy twice a week. These two patients were randomly allocated to receive either OT or placebo, twice a day for a period of 4 weeks. Both patients completed longitudinal assessments of process and outcome measures, and therapists' clinical notes were extracted and reviewed. Reliable clinical change was calculated for all outcome and process measures. The results indicated that the patient receiving OT showed significant improvement in interpersonal distress, as well as in anxiety and depression symptoms, while the placebo patient showed no significant change during the study period. Furthermore, while both patients showed no significant changes in the therapeutic alliance ratings, the therapist of the OT patient regularly reported positive changes in alliance in the medical notes, while no such report was observed in the placebo patient. These results suggest that changes produced by OT administration may be more noticeable by the therapist. Implications for future studies aimed at assessing the effect of OT on psychotherapy process and outcome are discussed.

## Introduction

Scholars have previously suggested that pharmacological aids such as oxytocin may be beneficial for the facilitation of psychotherapy (1). Oxytocin (OT) is a nine-amino acid neuropeptide synthesized in the magnocellular neurons of the paraventricular (PVN) and supraoptic nuclei (SON) of the hypothalamus and released by the pituitary gland (2). Often referred to as the “social bonding” hormone (3), studies have shown that OT may enhance trust and cooperative behaviors (4), enhance hypnotic responding (5), and expedite the formation of the therapeutic alliance between the therapist and the patient (6). Nonetheless, several experimental studies examining the effect of OT administration on psychotherapy outcomes have yielded contradicting results. For example, MacDonald et al. (7) found an increase in anxiety over the course of a single therapy session among depressed patients receiving intranasal administration of OT, compared to placebo. In another study, PTSD patients were randomly assigned to receive either OT or placebo prior to a weekly prolonged exposure therapy session, and found no statistically significant differences in depression, PTSD symptoms or alliance scores over the course of a 10-week treatment protocol (8). Although these studies utilized different methodological approaches, these differing results suggest that further clarification of the effect of OT on psychotherapy process and outcome is needed.

One of the approaches suggested to clarify an explored phenomenon is to perform an exploratory case-study (9, 10). Such an approach includes an in-depth account of the investigated phenomenon, so as to facilitate the refinement of the data collection process and to allow for a thoughtful and empirically guided hypotheses generation. Consistent with such objectives, this case report is aimed to provide an in-depth evaluation of the effect of OT administration on the processes and outcomes of psychotherapy, delivered in the settings of a psychiatric hospitalization. This evaluation was based on the first two patients recruited to an ongoing randomized, double-blind, placebo-controlled study, to evaluate the impact of IN-OT on psychotherapy process and outcomes (Clinical trial registration number - NCT03566069/MOH_2017-12-05_002003). The two patients were randomized to receive intranasal OT or placebo twice a day, for consecutive 28 days. Both patients were diagnosed with major depression and were hospitalized due to suicidal ideation. Both completed longitudinal assessments of process and outcome measures and were treated by therapists with similar orientation and training. Therapists' and nursing staff were blind to the allocation of the patients for each study condition. Clinical notes were reviewed to allow for a qualitative evaluation of the therapists' impressions of patients' interpersonal and emotional responsiveness during the sessions.

## Case Presentation

### Description of the Study Methods and Procedures

The study was set as an initial exploration aimed to assess feasibility and initial trends, following the initiation of a randomized, double-blind, placebo-controlled study, approved by the institutional review board (IRB) of Shalvata mental health center (IRB approval number: 0023-17-SHA). For the purposes of this case report, the first two cases randomized for each study condition were unblinded by the research staff, which were independent of the treating staff and had no prior acquittance with the participating patients. Thus, the first patient recruited to each study group was selected and analyzed. The two patients were admitted to psychiatric hospitalization due to depression. Psychiatric diagnoses were registered following a clinical interview and a review of the psychiatric patient file by the clinical staff of Shalvata MHC. Final diagnoses were assigned by treating psychiatrist using the diagnostic criteria of the DSM-V (11). After signing informed consent forms and approving their participation, the two patients were randomized and double-blindly allocated to receive either OT or placebo. Randomization was performed by an external nurse with no affiliation to the study. IN-OTplacebo was administered twice a day (at 08:00 a.m. and at 5:00 p.m.). The two patients were followed for 4 weeks. Psychotherapy was delivered twice a week. Throughout the intervention, both patients continued to receive their routinely prescribed psychiatric medication. Pre and post measurements included depression severity utilizing the Hamilton Rating Scale for Depression (HRSD) (12), anxiety levels using the State-Trait Anxiety Inventory (STAI) (13), attachment anxiety and avoidance using the Experience in Close Relationships scale (ECR) (14) and patient outcomes utilizing the Outcome Questionnaire-45 (OQ-45) (15). Working alliance was assessed bi-weekly using the Session Alliance Inventory (SAI) (16). The patient in the OT condition received 32IU (16IU*2) of OT, Sorbitol, Benzyl, alcohol glycerol, and distilled water. The patient in the control condition received 32IU (16IU*2) of Sorbitol, Benzyl, and alcohol glycerol. OT placebo was inhaled in two sprays, one in each nostril (8IU in each nostril). The dosage and form of delivery were determined according to standard guidelines in IN-OT studies (17). The ward staff were blinded to the substance administered to each patient.

### Description of Cases and Therapeutic Intervention

To allow for an in-depth evaluation of the effect of OT administration, we describe below the clinical background, therapy course, and extracts from therapists' clinical notes. We then provide an account of the changes in the longitudinal assessments filled by the patients, and later discuss their integration. All descriptions are completely disguised to allow for anonymity.

#### Patient 1: OT Administration Adjunct to Psychotherapy

***Clinical background***. Patient 1 (OT), is a 51-year-old single man, who is unemployed, lives alone, and is supported by social services. He was hospitalized in a psychiatric ward after expressing suicidal ideation. His current admission to the psychiatric ward followed a diagnosis of a depressive episode, with complaints of sadness and despair, decreased daily functioning, low appetite, and difficulty sleeping. This was his eighth admission.

***Therapy course***. As therapy was initiated, Patient 1 (OT) expressed a desire to work on his ongoing decrease in volition, stating that “my only wish is to start wanting again.” He mentioned his desire to regain occupational capacities and personal development. During the first 2 weeks of the intervention period, Patient 1 (OT) arrived late to the meetings, claiming he forgot the scheduled sessions. Observing this pattern, the patient and therapist discussed Patient1 (OT)'s ambivalence toward wanting to delve deeper into his inner world and explore it. The therapist also commented on Patient1 (OT)'s avoidant disposition, especially regarding close and intimate situations. Patient1 (OT) began to contemplate on his relationship with his former spouse, who often complained about his tendency to pull away “when things got tough.” In the following sessions, Patient1 (OT) shared his disappointment that his ex-spouse didn't “see him and accept him for who he was,” not showing him the warmth and support for which he longed. Toward the end of the intervention period, Patient1 (OT) began to arrive on time, wearing his own clothes instead of hospital garments. The therapist noted these changes, while also observing that he had become more active, engaged and involved in their sessions, as well as in the overall treatment course.

***Therapist's clinical notes***. After Patient1 (OT) was recruited into the study, the therapist made the following observations in the clinical notes: “the patient was very communicative and pleasant”; “the patient was more expressive and his affect display was broader than usual”; “he seemed more present and engaged in what was said in the session.” In another session, the therapist remarked that the patient “seemed more attuned and attentive than before.”

#### Patient 2: Placebo Administration Adjunct to Psychotherapy

***Clinical background***. Patient 2 (PLC), is a 60 years old divorced man, who has two adult daughters, and lives alone in sheltered housing. He is unemployed and is supported by social services. His current admission to the psychiatric ward followed a diagnosis of a depressive episode, with complaints of depressive symptoms such as feelings of sadness and despair, inability to function, ongoing isolation, cessation of eating, and suicidal thoughts. This was his third admission.

***Therapy course***. In the beginning, the main goals of the therapy were to focus on concrete issues regarding alternative sheltered housing and occupational setting options. During sessions, Patient 2 (PLC) discussed his relationship with his siblings, his feelings of inferiority, and his ambivalence toward his family's financial support, realizing the emotional cost of being in an unsteady relationship with them. He often shared with the therapist his feelings of loneliness and sadness. In the last sessions of the intervention period, after receiving bad news regarding housing options, he expressed frustration and disappointment in his therapist, as well as with the psychiatric system which was unable to provide him with the assistance he needed.

***Therapist's clinical notes***. After Patient2 (PLC) was recruited into the study, the therapist made the following observations in the clinical notes: “the patient made minimal eye contact and didn't seem engaged in the conversation”; “the patient's affect display was flat and unchanging throughout the session"; Patient2 (PLC) “seemed gloomy, lethargic, and uninterested in what was said.” In another session, the therapist remarked that the patient “seemed detached and withdrawn, especially when discussing his difficulties with past and present relationships.” In the last sessions during the intervention period, the therapist noted that “the patient seemed overwhelmed with sadness.”

### Changes in Outcome and Process Measures

To compare the clinical trajectories of the two cases, we utilized the Jacobson and Truax (18) approach to reliable clinical change, which includes a calculation of the Reliable Change Index (RCI >1.96) and a return to functional distribution or out of a dysfunctional distribution (SD>2.0). Normative and clinical cutoffs utilized to determine the RCI in each measure is elaborated in the Supplementary Material (see Supplementary Table 1).

Table 1 presents the pre- and post-treatment scores for both patients, as well as the reliable clinical change index scores.

**Table 1:** Mean scores of levels of anxiety, depression, interpersonal relations (distress), attachment avoidance and attachment anxiety, by time of measurement in the two clinical demonstrations comparing Patient 1, receiving IN-OT, vs. Patient 2, receiving PLC.

	**Patient 1 (OT)**			**Patient 2 (PLC)**		
**Measure**	**Pre**	**Post**	**RCI**	**Pre**	**Post**	**RCI**
Anxiety (STAI-S)	55.00	43.00^a^^,^^a#^	3.23	79.00	80.00	−0.26
Depression (HRSD)	20.00	18.00^a^	2.02	22.00	30.00^b^	−8.09
Symptom distress (OQ-45, SD Subscale)	42.00	42.00	0.00	78.00	72.00	1.06
Interpersonal distress (OQ-45, IR Subscale)	21.00	16.00^a#^	1.22	20.00	32.00^b^	−2.94
Social role (OQ-45, SR Subscale)	12.00	7.00	1.28	12.00	8.00	1.03
Attachment avoidance (ECR)	4.67	4.00^a#^	1.49	6.67	6.83	−0.36
Attachment anxiety (ECR)	2.94	4.00^b^	−2.06	6.38	6.33	0.09
Working alliance (SAI)	4.44	4.00	1.14	6.66	6.50	0.43

*STAI-S, State-Trait Anxiety Inventory; HRSD, Hamilton Rating Scale for Depression; OQ-45, Outcome Questionnaire-45; SD, Symptom Distress subscale; IR, Interpersonal Relations subscale; SR, Social Role subscale; ECR, Experience in close relationship scale; SAI, Session Alliance Inventory. Effect sizes were calculated between pre-treatment scores (t1) and post-treatment scores (t2)*.

a *Clinically significant change (improvement) according to Jacobsen & Truax (18): RCI > 1.96*.

b *Clinically significant change (deterioration)*.

a# *movement from dysfunctional to functional distribution*.

**Figure 1:**
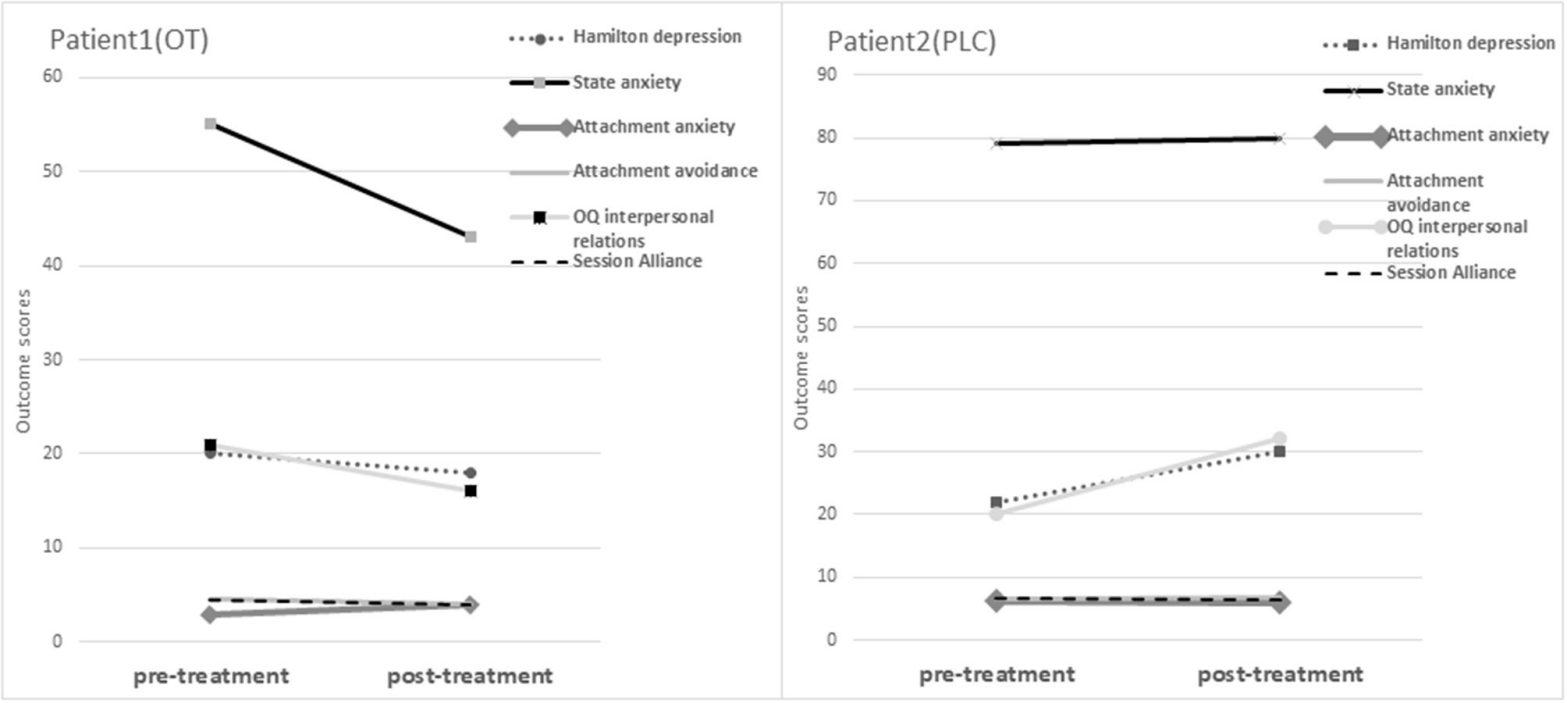
Changes in outcomes in Patient 1 (OT) and Patient 2 (PLC) reports.

As can be viewed, a significantly reliable improvement was noted in Patient 1 (OT)'s HRSD, indicating an improvement in depression symptoms and severity from pre-treatment to post-treatment. On the other hand, Patient 2 (PLC)'s scores indicated an increase in depression symptoms, with a statistically significantly reliable deterioration in the HRSD score. There was a significantly reliable improvement and movement from clinical to functional distribution in Patient 1 (OT)'s scores on the STAI-S, indicating a significant improvement in anxiety symptoms from pre- to post-intervention. Conversely, Patient 2 (PLC)'s report indicated that no reliable change on the STAI-S. On the OQ-45, no significant reliable change was detected on either the SD or the SR indexes, for either Patient 1 (OT) or Patient 2 (PLC). However, a movement from clinical to functional distribution was evident in Patient 1 (OT)'s IR index scores, indicating a decrease in interpersonal relations distress, yet this trend wasn't statistically significant. In contrast, Patient 2 (PLC)'s scores indicated an increase in symptoms, with a significant deterioration on the IR scale, indicating that his interpersonal relations distress increased from pre- to post-treatment. On the attachment scales, a significantly reliable deterioration was noted on Patient 1 (OT)'s ECR anxious attachment scale, indicating that attachment anxiety increased from pre- to post-treatment. On the avoidance scale, a movement from clinical to functional distribution was evident in Patient 1 (OT)'s scores, indicating a slight decrease in avoidant attachment; however, this trend wasn't reliably significant. In comparison, there was no movement evident in Patient 2 (PLC)'s scores on the avoidance scale. On the SAI, no significant reliable change was detected for either Patient 1 (OT) or Patient 2 (PLC) and there was no movement evident from clinical to functional distribution. Figure 1 presents a visual representation of the changes in outcome measures across the two study participants.

## Discussion

This study was aimed to explore the effect of OT administration on psychotherapy processes and outcomes using a case study methodology. The results indicated that Patient 1 (OT) showed improvements in anxiety and depression, and a decrease in interpersonal distress, during the course of 1-month of intervention. Furthermore, Patient 1 (OT) reported a decrease in attachment avoidance, while also showing an increase in attachment anxiety. There were no apparent improvements on either the symptom distress or social role self-report indexes of the OQ-45. Conversely, Patient 2 (PLC) demonstrated no improvement in either depression or anxiety and reported an increase in interpersonal distress, with no change in the attachment scales or in the symptom distress and social role indexes of the OQ-45. Neither patients showed a significant change in alliance; however, Patient 1 (OT)'s therapist clinical notes clearly indicated a positive trend of more engagement and attunement in the sessions. Although the interpretation of the results should take the case report methodology into account, the findings of this exploratory case study point to several potential considerations which might inform future initiatives aimed at assessing the effect of OT administration on psychotherapy.

The reductions in depression and anxiety symptoms and improvement in interpersonal relations in the patient receiving OT resonate with previous findings demonstrating a beneficial effect of OT on psychotherapy outcome when given adjacent to psychotherapy (19, 20). As the working alliance is considered the most robust predictor of therapy outcome (21), one potential hypothesis that can be made is that OT affects the outcome indirectly through the facilitation of the therapeutic process. Nonetheless, it should be acknowledged that studies also suggest that OT has an antidepressant-like effect, and can therefore have a direct effect on the reduction in depressive symptoms (22, 23). It has also been suggested that symptomatic improvements often observed in IN-OT clinical trials may be attributed to improvements in social functioning (24), social cognition (25), and theory of mind (7, 26). Although these linkages could not be assessed in this case report study, the participant receiving IN-OT indeed demonstrated a decrease in interpersonal distress, which can be associated with increased social functioning. These hypotheses should be explored in future studies assessing potential mediators of the OT effect on symptomatic relief.

Both patients showed no significant changes in alliance patterns throughout the study, nonetheless, the therapists of the OT patient repeatedly reported an improvement in patient's engagement. One potential explanation to account for these findings is that potential deficits in social cognition, which are usually eminent during periods of severe depression (27), made it difficult to identify micro-changes in interpersonal connection in short time periods. In a parallel manner, Bryant et al. (5) also found that IN-OT improved the level of hypnotic responding in 40 low-hypnotizable healthy male subjects, yet it did not alter patients' self-reports of trust. Thus, it can be hypothesized that the interpersonal effect of OT is too nuanced to be detected by patients, especially with patients suffering from severe pre-treatment social deficits. This potential explanation might indicate that future initiative should explore both patient and therapist rankings of the alliance, so as to capture these micro changes.

Patients' changes in attachment patterns may also demonstrate an important effect of the OT which should be subjected to further exploration. The results indicated that Patient 1 (OT) had an improvement in avoidant attachment and a deterioration in anxiety attachment, while Patient 2 (PLC) showed no significant changes in either factors. Previous studies have argued that OT reduces avoidance and increases approach behaviors (28). Therefore, while OT diminishes avoidant patterns, it may simultaneously amplify interpersonal insecurities among those anxiously attached (29). Hence, it is possible that the decline in avoidance reported in the OT patient, lead to the activation of anxiously-attached patterns (30). Future studies should further explore whether such a pattern may be associated with the effect of OT administration.

The findings reported in this case report led to important directions for future research, and can assist in refining research hypotheses and designs for studies attempting to elucidate the effect of OT on psychotherapy process and outcome. The pattern of processes and outcomes presented in the two case reports point to the need to direct future research to the effect of OT on therapy processes as reported by both patients and therapists, as well as to explore social and interpersonal mediators of these effects. Clinically, the results suggest that OT can potentially act as an agent for a rapid psychotherapeutic response, which can be crucial for inpatient therapeutic interventions. Such direction is critical in psychiatric hospitals, which sometimes utilize the augmentation of psychiatric medications to elicit a rapid therapeutic response (31). Several limitations should nonetheless be acknowledged. As this study utilized a case report methodology, baseline clinical and demographic differences between the two cases (e.g., marital status, or the presence of physical and neurodevelopmental comorbidities), may have influenced the reported differential course and outcome. Thus, causal interpretations cannot be assumed, and future studies should further examine our results while using larger matched samples. Moreover, the two patients reported in this study were treated by different therapists, thus, therapists' effects may account for the potential differences in process and outcome. Finally, the utilization of therapists' medical notes was greatly informative, nonetheless, they were not accompanied by a standardized tool to quantitatively assess systematic differences between the reports. Notwithstanding these limitations, the current case illustrations provide important insights for the exploration of the effect of OT in natural clinical settings, which can potentially serve both research and clinical practice.

## Data Availability Statement

The datasets presented in this article are not readily available due to ethical restrictions. Requests to access the datasets should be directed to danatz@ariel.ac.il.

## Ethics Statement

The study was approved by the Shalvata Mental Health Center (MHC) institutional review board (IRB Approval No: 0023-17-SHA). The patients/participants provided their written informed consent to participate in this study.

## Author Contributions

AG-G performed the literature review, collected, analyzed and interpreted the data, and drafted the manuscript. DTB initiated the study and critically revised the manuscript. SZ-M participated in data analysis and interpretation and critically revised the manuscript. UN and SM participated in data collection and critically revised the manuscript. HM initiated the study, participated in data collection and management, interpreted the data, and critically revised the manuscript. All authors contributed to the article and approved the submitted version.

## Funding

This project was supported by a grant from the American Psychological Foundation.

## Conflict of Interest

The authors declare that the research was conducted in the absence of any commercial or financial relationships that could be construed as a potential conflict of interest.

## Publisher's Note

All claims expressed in this article are solely those of the authors and do not necessarily represent those of their affiliated organizations, or those of the publisher, the editors and the reviewers. Any product that may be evaluated in this article, or claim that may be made by its manufacturer, is not guaranteed or endorsed by the publisher.
