# Supplementary material for: Case Report: Oxytocin and Its Association With Psychotherapy Process and Outcome
Source: Front Psychiatry. 2021 Sep 14;12:691055. doi: 10.3389/fpsyt.2021.691055 (PMC8477032; doi:10.3389/fpsyt.2021.691055)
Supplement: Supplementary file 1 [file Data_Sheet_1.docx]

**Supplementary Material**

**Statistical strategy**

To compare the clinical trajectories of the two cases, we utilized the Jacobson & Truax (1991) approach to reliable clinical change, which includes a calculation of the Reliable Change Index (RCI >1.96) and a return to functional distribution or out of a dysfunctional distribution (SD>2.0). Normative and clinical data for the HRSD were obtained from a study assessing the development of a computer-administered version of the HRSD (Kobak, Reynolds, & Greist, 1993), which reported a mean of 2.4 (SD = 2.47) for the normative sample, 20.31 (SD = 6.03) for the clinical sample, and a Cronbach’s alpha of 0.92. STAI-S measures were obtained from a study assessing the psychometric properties of an anxiety measure in a non-clinical sample (Creamer, Foran, & Bell, 1995), and from a study assessing the psychometric properties of a scale measuring depression and anxiety in a patients suffering from depression (Gorenstein & Andrade, 1996). These studies reported a mean score of 38.5 (SD = 10.7) for normative populations, 56.4 (SD = 10.5) for clinical populations, and a Cronbach’s alpha of 0.94. Normative measures of the OQ‐45 were obtained from a study assessing a cross‐cultural validation of the OQ-45 (De Jong et al., 2007). Clinical data were obtained from a study assessing the efficacy of the OQ-45 with feedback among a psychiatric outpatient population (Hansson, Rundberg, Österling, Öjehagen, & Berglund, 2013). Cronbach’s alpha for the Symptom Distress index was 0.89 (mean 25.4±12.0 for the normative sample and 55.3±12.9 for the clinical sample); for the Interpersonal Relations index 0.77 (mean10.2±6.0 for the normative sample and 21.3±3.0 for the clinical sample); and for the Social Role index 0.53 (mean 9.6±4.0 for the normative sample and 15.4±4.1 for the clinical sample). Normative data for the ECR were obtained from a study reporting its psychometric properties (Conradi, Gerlsma, Van Duijn, & De Jonge, 2006), while clinical data was obtained from a study assessing the reliability of the measure in patients with severe psychopathology (Picardi, Martinotti, Paci, Simi, & Caroppo, 2011). For the avoidant attachment scale of the ECR, the Cronbach’s alpha was 0.88 (mean 2.49±0.91 for the normative sample and 53.9±21.9 for the clinical sample). For the anxious attachment scale, the Cronbach’s alpha was 0.86, and the means were 3.09±0.97 for the normative sample and 86.8±20.4 for the clinical sample. Data for the SAI was obtained from the original study developing the the SAI instrument (Falkenström et al., 2015). Data of the normal sample was extracted from sample 1 which comprised patients with no formal diagnoses. Data for the clinical sample was extracted from sample 3, comprised of patients from psychiatric units. In their study (Falkenström et al., 2015), The Cronbach’s alpha was 0.94, and the means were 5.95±1.11 for the normative sample and 5.87±1.20 for the clinical sample.

Table S1. Sources, normative and clinical data (Mean scores, SD, and Alpha coefficient) utilized for calculations of RCI's of measures of anxiety, depression, symptom distress, social role, interpersonal relations (distress), attachment avoidance and attachment anxiety.

|  | Normative samples | | | Clinical samples | | Sources | |
| --- | --- | --- | --- | --- | --- | --- | --- |
| Measure | Mean | SD | Alpha | Mean | SD | Non-clinical sample | Clinical sample |
| Anxiety (STAI-S) | 38.5 | 10.7 | 0.94 | 56.4 | 10.5 | Creamer M, Foran J, Bell R. The Beck Anxiety Inventory in a non-clinical sample. Behav Res Ther (1995) 33:477-85. doi:10.1016/0005-7967(94)00082-U | Gorenstein C, Andrade LH. Validation of a Portuguese version of the Beck Depression Inventory and State-Trait anxiety inventory in Brazilian subjects. Braz J Med Biol Res (1996) 29:453–57. |
| Depression (HRSD) | 2.4 | 2.47 | 0.92 | 20.31 | 6.03 | Kobak KA, Reynolds WM, Greist JH. Development and validation of a computer-administered version of the Hamilton Rating Scale. Psychol Assess (1993) 5:487. doi:10.1037/1040-3590.5.4.487 | Kobak KA, Reynolds WM, Greist JH. Development and validation of a computer-administered version of the Hamilton Rating Scale. Psychol Assess (1993) 5:487. doi:10.1037/1040-3590.5.4.487 |
| Symptom Distress (OQ-45) | 25.4 | 12 | 0.89 | 55.3 | 12.9 | de Jong K, Nugter MA, Polak MG, Wagenborg JE, Spinhoven P, Heiser WJ. The Outcome Questionnaire (OQ‐45) in a Dutch population: A cross‐cultural validation. Clin Psychol Psychother (2007) 14:288-301. doi:10.1002/cpp.529 | Hansson H, Rundberg J, Österling A, Öjehagen A, Berglund M. Intervention with feedback using Outcome Questionnaire 45 (OQ-45) in a Swedish psychiatric outpatient population. A randomized controlled trial. Nord J Psychiatry (2013) 67:274-81. doi:10.3109/08039488.2012.736534 |
| Interpersonal Distress (OQ-45) | 10.2 | 6 | 0.77 | 21.3 | 3 | de Jong K, Nugter MA, Polak MG, Wagenborg JE, Spinhoven P, Heiser WJ. The Outcome Questionnaire (OQ‐45) in a Dutch population: A cross‐cultural validation. Clin Psychol Psychother (2007) 14:288-301. doi:10.1002/cpp.529 | Hansson H, Rundberg J, Österling A, Öjehagen A, Berglund M. Intervention with feedback using Outcome Questionnaire 45 (OQ-45) in a Swedish psychiatric outpatient population. A randomized controlled trial. Nord J Psychiatry (2013) 67:274-81. doi:10.3109/08039488.2012.736534 |
| Social Role (OQ-45) | 9.6 | 4 | 0.53 | 15.4 | 4.1 | de Jong K, Nugter MA, Polak MG, Wagenborg JE, Spinhoven P, Heiser WJ. The Outcome Questionnaire (OQ‐45) in a Dutch population: A cross‐cultural validation. Clin Psychol Psychother (2007) 14:288-301. doi:10.1002/cpp.529 | Hansson H, Rundberg J, Österling A, Öjehagen A, Berglund M. Intervention with feedback using Outcome Questionnaire 45 (OQ-45) in a Swedish psychiatric outpatient population. A randomized controlled trial. Nord J Psychiatry (2013) 67:274-81. doi:10.3109/08039488.2012.736534 |
| Attachment Avoidance (ECR) | 2.49 | 0.91 | 0.88 | 53.9 | 21.9 | Jan Conradi H, Gerlsma C, Duijn MV, Jonge PD. Internal and external validity of the experiences in close relationships questionnaire in an American and two Dutch samples. Eur J Psychiatry (2006) 20:258-69. doi:10.4321/s0213-61632006000400006 | Picardi A, Martinotti G, Paci M, Simi C, Caroppo E. Reliability of self-reported attachment style in patients with severe psychopathology. J Pers Assess (2011) 93:491-9. doi:10.1080/00223891.2011.594128 |
| Attachment Anxiety (ECR) | 3.09 | 0.97 | 0.86 | 86.8 | 20.4 | Jan Conradi H, Gerlsma C, Duijn MV, Jonge PD. Internal and external validity of the experiences in close relationships questionnaire in an American and two Dutch samples. Eur J Psychiatry (2006) 20:258-69. doi:10.4321/s0213-61632006000400006 | Picardi A, Martinotti G, Paci M, Simi C, Caroppo E. Reliability of self-reported attachment style in patients with severe psychopathology. J Pers Assess (2011) 93:491-9. doi:10.1080/00223891.2011.594128 |
| Working Alliance (SAI) | 5.95 | 1.11 | 0.94 | 5.87 | 1.2 | Falkenström F, Hatcher RL, Skjulsvik T, Larsson MH, Holmqvist R. Development and validation of a 6-item working alliance questionnaire for repeated administrations during psychotherapy. Psychol Assess (2015) 27:169-83. doi:10.1037/pas0000038 | Falkenström F, Hatcher RL, Skjulsvik T, Larsson MH, Holmqvist R. Development and validation of a 6-item working alliance questionnaire for repeated administrations during psychotherapy. Psychol Assess (2015) 27:169-83. doi:10.1037/pas0000038 |

*Notes*. STAI-S = State-Trait Anxiety Inventory; HRSD = Hamilton Rating Scale for Depression;OQ-45 = Outcome Questionnaire-45, SD = Symptom Distress subscale, IR = Interpersonal Relations subscale, SR = Social Role subscale; ECR = Experience in close relationship scale; SAI = Session Alliance Inventory.
